# Persistence and mobility of metazachlor and its metabolites in soils: Why transformation products pose a greater groundwater risk

**DOI:** 10.1007/s10653-026-03452-w

**Published:** 2026-09-02

**Authors:** Radka Kodešová, Daniela Tomešová, Martin Kočárek, Miroslav Fér, Aleš Klement, Antonín Nikodem, Jindřich Zelinka, Vít Kodeš

**Affiliations:** 1 Department of Soil Science and Soil Protection, Faculty of Agrobiology, Food and Natural Resources, Czech University of Life Sciences Prague, Kamýcká 129, 16500 Prague 6, Czech Republic; 2 Project Department, ALS Czech Republic, Na Harfě 336/9, 19000 Prague 9, Czech Republic; 3 Czech Hydrometeorological Institute, Na Šabatce 2050/17, 14306 Prague 4, Czech Republic

**Keywords:** Sorption isotherms, Half-lives, Transformation of compounds, Soil properties, Prediction of compound behavior in soils, Metabolite yield

## Abstract

Metazachlor is a widely used herbicide that is transformed into various metabolites that are often found in groundwater. To assess their mobility and persistence in the soil environment, batch experiments were performed for the parent compound and two most frequently occurring metabolites OA (479M04) and ESA (479M08), using representative soils of the Czech Republic. In addition, the concentrations of all three compounds and two additional metabolites (479M09 and 479M11) were measured during metazachlor degradation experiments to determine the formation and dissipation rates of all four metabolites.

Metazachlor sorbed more strongly and was less persistent than OA and ESA. The sorption of all compounds was positively influenced by the soil organic carbon content, as well as by the sand and silt content, and hydrolytic acidity (metabolites). Dissipation was positively affected by soil organic carbon and clay content and in some cases negatively by sorption. Metazachlor, and particularly OA and ESA, exhibited lower sorption and higher stability in soil than the published values indicate. Even higher stability of four metabolites was observed during the metazachlor transformation. The dissipation of 479M09 (half-live 15–111 days) and 479M11 (39–87) was faster than that of OA (18–492) and ESA (110–231) but much slower than metazachlor dissipation (7–17). The combined formation fractions of four metabolites derived from metazachlor ranged from 60 to 95%. Notably, the yields of OA (27–47%) and potentially carcinogenic 479M09 (10–25%) were significantly higher than database values, suggesting a greater environmental risk than expected.

**Supplementary Information:**

The online version contains supplementary material available at https://doi.org/10.1007/s10653-026-03452-w.

## Introduction

When studying the behavior of pesticide substances, i.e. their sorption and degradation, in the soil environment, attention is usually focused on the parent compounds. Those that are strongly sorbed in the soil and degrade relatively quickly are considered relatively safe because they remain where they are intended to act, thus minimizing the risk of aquatic environment contamination. However, the degradation of these substances often produces metabolites that can persist in the environment for a much longer duration. Additionally, these metabolites are often less strongly sorbed to soil and sediment, making them much more mobile in the environment and increasing the risk of groundwater contamination. An example is metazachlor and its metabolites metazachlor OA (479M04) and metazachlor ESA (479M08), which are frequently found in both surface and groundwater (Baran et al., 2022; Cooke et al., 2024; CHMI, 2025; Halbach et al., 2021; Hvězdová et al., 2018; Karier et al., 2017; Kodeš et al., 2023; Kodeš, 2020; LAWA, 2019; Lischeid et al., 2023; Reemtsma et al., 2013; Sadchenko et al., 2026; Uber et al., 2025; Ulrich et al., 2021) and have also been identified in drinking water (NIPH, 2025; Ulrich et al., 2021). Other metabolites of metazachlor that may be formed in the soil environment are metabolites 479M09, 479M11, and 479M12 (Lewis et al., 2016). All three metabolites can be found in surface water and sediments, and metabolites 479M09 and 479M11 can be very scarcely found in groundwater (Kiefer et al., 2019; LAWA, 2019; Reemtsma et al., 2013; Sur et al., 2024). When plants absorb parent compound and metabolites formed in soil via roots (alternatively via foliage), organism-specific enzymatic processes can transform these compounds into additional metabolites, such as 479M16 (APVMA, 2016; Park et al., 2020). While metabolites 479M09 and 479M11 are considered potentially toxic (carcinogenic), metabolites OA (479M04) and ESA (479M08) are considered toxicologically irrelevant (EFSA, 2008, 2017a; Lewis et al., 2016). On the other hand, according to Lewis et al. (2016), metabolites 479M9 and 479M11 are assumed to form only a minor fraction of the parent compound compared to the metabolites 479M4 (OA) and 479M8 (ESA).

Knowledge regarding the behavior and environmental impact of metazachlor and its metabolites has been summarized, for example, in the Pesticide Properties DataBase–PPDB (Lewis et al., 2016) or by EFSA (2008, 2017a) and APVMA (2016). While there is a lot of information for the parent compound, there is much less information for the OA and ESA metabolites, and even less for other metabolites. Moreover, these databases do not include, apart from the Koc values, which express the relationship between sorption affinity and the organic matter content, other possible relationships of sorption affinity or transformation rates of the parent compound and its metabolites to soil properties. It has been shown for a wide range of organic micropollutants that the sorption of polar micropollutants is also controlled by mechanisms other than those associated with organic matter (e.g., Kodešová et al., 2011, 2023, 2026b). Similarly, dissipation of organic compounds strongly depends on soil conditions (e.g., Kodešová et al., 2020, 2026a).

Therefore, this study aimed to comprehensively evaluate the sorption and dissipation of metazachlor and its two metabolites, OA and ESA, across eight (sorption) and five (dissipation) representative soils, while correlating these characteristics with soil properties. A further objective was to compare the dissipation rates of both metabolites when applied directly to the soil versus their degradation rates during the transformation of the parent compound. Additionally, the study aimed to determine the dissipation rates of two other metabolites, 479M09 and 479M11, formed alongside OA and ESA. Finally, a further key objective was to quantify the formation fractions of all four metabolites to test the hypothesis that 479M09 and 479M11 are minor transformation products compared to OA and ESA. Information on the production of these minor metabolites and their stability can help to assess whether they may pose a higher environmental threat.

## Materials and methods

### Metazachlor and its metabolites

Metazachlor (2-chloro-N-(2,6-dimethylphenyl)-N-(1H-pyrazol-1-ylmethyl)acetamide) and its metabolites, metazachlor oxanilic acid (metazachlor OA, 479M04, N-(2,6-dimethylphenyl)-N-(1H-pyrazol-1-ylmethyl)oxalamide) and metazachlor ethanesulfonic acid (metazachlor ESA, 479M08, N-(2,6-dimethylphenyl)-N-(1H-pyrazol-1-ylmethyl)aminocarbonylmethylsulfonic acid), were included among the target analytes (Table S1). In degradation experiments of all three compounds, the transformation products 479M09 (N-(2, 6-dimethylphenyl)-N-(1H-pyrazol-1-ylmethyl)aminocarbonylmethylsulfinyl acetic acid) and 479M11 (methyl N-(2,6-dimethylphenyl)-N-(1H-pyrazol-1-ylmethyl)aminocarbonylmethylsulfoxide) were also monitored (Table S1). Analytical standards for all these compounds and internal standards were purchased from Neochema (Bodenheim, Germany). Analytical standards for performing sorption and degradation experiments were purchased from LGC Labor GmbH (Augsburg, Germany). Acetonitrile (LC/MS grade, Sigma-Aldrich), sodium chloride (NaCl, p.a., Lach-ner), magnesium sulfate (MgSO_4_, >99.5%, Sigma-Aldrich), sodium citrate dibasic sesquihydrate (Na_2_C_6_H_5_O_7_. 1.5H_2_O, >99%, Sigma-Aldrich), sodium citrate tribasic dihydrate (Na_3_C_6_H_5_O_7_. 2H_2_O, ACS reagent, Sigma-Aldrich) were used for sample preparation. Ammonium acetate (LC/MS, >99%, Sigma-Aldrich) and formic acid (for LC/MS, 98%, Lach-ner), Ultrapure Milli-Q water (Milli-Q system, Millipore, Darmstadt, Germany), and methanol (LC/MS grade, Sigma-Aldrich) were used throughout the study.

### *Soilsandsoilproperties*

To investigate the sorption and transformation of metazachlor and its metabolites in soils, eight distinct soil types were selected (Table S2): Chernozem Siltic on marlite (SChS), Haplic Chernozem on loess (HCh), Greyic Phaeozem on loess (GP), Haplic Luvisol on loess (HL), two Haplic Cambisols on paragneiss (HCa–A and HCa–B), Dystric Cambisol on orthogneiss (DCa), and Arenic Regosol on sand (AR). Representing a broad spectrum of Central European soil conditions, this selection allows the resulting data and statistical analyses to be applied to the interpretation of compound behavior (i.e., sorption and transformation) across the Czech Republic and surrounding countries with similar climate and soil substrates. Soil samples for the laboratory experiments were collected from a 0–25 cm depth, air-dried, and sieved to <2 mm. Under constant laboratory conditions (20 °C), the following physico-chemical properties were measured: active soil reaction (pH_H2O_) and potential soil reactions (pH_KCl_, pH_CaCl2_) (ISO, 10,390:2005), oxidizable organic carbon content (C_ox_) (Skjemstad and Baldock, 2008), CaCO_3_ content (Loeppert and Suarez, 1996), salinity (Rhoades 1996), particle density (ρ_s_) (Flint and Flint, 2002), particle size distribution (Gee and Or 2002), cation exchange capacity (CEC) (Bower and Hatcher 1966), and hydrolytic acidity (HA) (Klute, 1996). Additionally, base cation saturation (BCS) was calculated as the difference between CEC and HA, and sorption complex saturation (SCS) was expressed as the percentage of BCS in CEC. Correlations between soil properties are shown in Table S3.

### *Sorptionexperiment*

Sorption isotherms for metazachlor and its two metabolites OA and ESA were determined using the standard batch equilibrium method (OECD, 2000), following the protocol established in our previous studies (Kodešová et al., 2011, 2023; Schmidtová et al., 2020). Triplicate samples were prepared in disposable plastic centrifugation tubes using 10 g dry soils and 20 cm^3^ solution 0.01 M CaCl_2_ with five compound concentrations (0.5, 1, 2, 5, or 10 µg cm^−3^), which corresponds to probable concentration in soil water after compound application. For example, if considering a recommended application concentration of the solution (1000 µg cm^−3^), the application rate of metazachlor (0.5 kg per hectare, i.e., 5 μg cm^−2^, APVMA, 2016), soil water content (0.25 cm^3^ cm^−3^, e.g., Pavlů et al., 2021; Thet et al., 2026), and uniform compound distribution to a depth of 3 cm, then the concentrations in the soil solution will be 6.3 µg cm^−3^. Tubes were shaken on a reciprocating shaker (GFL 3006, Gesellschaft für Labortechnik mbH) at 20 °C for 24 h to reach equilibrium (Kodešová et al., 2023). Following centrifugation (10 min, 6000 rpm) and filtration (0.45 μm regenerated cellulose filters, Labicom, Olomouc, Czech Republic), supernatants were immediately analyzed. Solution-only controls (collected during dosing and treated in the same manner) confirmed that the plastic tubes and filters did not affect the results.

The actual initial concentrations, C_ini_ (μg cm^−3^), and final equilibrium concentrations, C_eq_ (μg cm^−3^), were quantified using a direct injection procedure. Each sample was diluted as needed according to the expected analyte concentration. After addition of the internal standard, the samples were filtered through 0.2 µm cellulose membrane filters and transferred to autosampler vials for LC–MS analysis. All target analytes were determined by ultra-performance liquid chromatography coupled with tandem mass spectrometry (UPLC-MS/MS). Chromatographic separation was performed using an Acquity UPLC I-Class system (Waters, Milford, MA, USA) equipped with an Acquity UPLC BEH C18 analytical column (1.7 µm particle size, 2.1 × 100 mm; Waters, Milford, MA, USA). Depending on an instrumental method, the column temperature was maintained between 40 °C (metazachlor) and 65 °C (metabolites ESA and OA), and injection volume of samples was 100 µL. Gradient elution was applied at a mobile phase flow rate of 0.45 ml L^−1^. As a mobile phase 5 mM ammonium acetate for metazachlor or 5 mM ammonium acetate with 0.01% formic acid for metabolites ESA and OA (A) and methanol (B) were used. Mass spectrometric detection was carried out using XEVO TQ-S and XEVO TQ-XS tandem mass spectrometers (Waters, Milford, MA, USA) equipped with an electrospray ionization (ESI) source operating in both positive and negative ionization modes. The principal instrument settings were as follows: capillary voltage 1500 V; cone voltage adjusted according to analyte-specific method requirements (listed in Table S4); desolvation temperature 500 °C; source temperature 150 °C; and desolvation gas flow 1100 L h^−1^. Target analytes were identified on the basis of their characteristic multiple reaction monitoring (MRM) transitions and retention times (listed in Table S4). Isotopically labeled internal standards were applied for quality control of analyses and final quantification of compounds. The UPLC-MS/MS system was controlled using MassLynx software (v. 4.2; Waters), the data were processed using Target Lynx software (v. 4.2; Waters) with additional Excel data processing. Detailed information on LC–MS/MS methods, including the LOQ range for the given analytes, is provided in the supplementary materials (Table S4).

The sorbed compound concentration, s (µg g^−1^), was calculated as follows:

1 $$s=\frac{V({{c}_{ini}-c}_{eq})}{m}=2({{c}_{ini}-c}_{eq})$$

where V is the volume of applied solution (20 cm^3^) and m is the mass of dry soil (10 g). The experimental sorption isotherm data, i.e., the equilibrium concentrations (c = c_eq_) paired with s values, were fitted using the Freundlich equation:

2 $$s = K_{F} c^{{{\raise0.7ex\hbox{$1$} \!\mathord{\left/ {\vphantom {1 n}}\right.\kern-0pt} \!\lower0.7ex\hbox{$n$}}}}$$

where K_F_ (cm^3/n^ μg^1−1/n^ g^−1^) and n (–) are empirical coefficients. Logarithmic transformation was applied to the data and Eq. (2) (Jorgensen and Bendorichio, 2001), followed by the determination of unknown coefficients via the least squares method (Table S5, Figure S1). Because K_F_ values are dependent on n values, directly using them to assess soil properties’ influence on sorption affinity is mathematically unsound. Therefore, consistent with our previous studies (e.g., Kodešová et al., 2023, 2026b; Schmidtová et al., 2020), the average coefficient n_avg_ was calculated for each compound across all eight soils. Then a new set of K_F_ values was calculated by fitting the experimental data with Eq. (2) with fixed n at n_avg_ (Tables S5 and 1, Figure S1).

These values were subsequently used for statistical analyses. As reported by Schmidtová et al. (2020) and Kodešová et al. (2023), although this approach suppresses information about the curvature of the sorption isotherm for each soil, the K_F_ and n_avg_ values describe general trends in the sorption isotherms and allow the development of models for predicting the sorption behavior of compounds in different soils.

### *Degradationexperiment*

Dissipation rates and half-lives of metazachlor and its two metabolites were evaluated using the batch degradation method (OECD, 2002). For each soil type and compound, 27 high-density polyethylene 100 mL bottles were prepared. Each bottle received 50 g of air-dried soil, which had been dried, ground, and sieved within a maximum of 5 days. To ensure optimal microbial conditions, i.e., avoiding potential impairment from air-drying (OECD, 2002), 6 cm^3^ of fresh water (3 cm^3^ for Regosol) was added, and the samples were incubated in the dark at a constant 20 °C. Following the initial moistening, 6 cm^3^ (4 cm^3^ for Regosol) of compound solution was added to the soil, and incubation continued. The water additions were calculated to target approximately one-third (step 1) and two-thirds (step 2) of the water-holding capacity, respectively. Solution concentrations were adjusted to ensure a consistent load of 1 μg g^−1^ of dry soil, which again roughly corresponds to the application rate of metazachlor (0.5 kg per hectare, i.e., 5 μg cm^−2^, APVMA, 2016). Assuming the soil bulk density of 1.5 g cm^−3^ and uniform compound distribution to a depth of 3 cm, then the concentrations in the soil will be 1.1 µg g^−1^. The following concentrations of applied solutions were assumed: 8.3 μg cm^−3^ (SChS, HCh, HCa-A, and HCa-B) and 12.5 μg cm^−3^ (AR). Water and solution volumes were precisely determined by weighing each bottle at 4 stages: empty, with soil, after adding water, and finally after adding solution. Throughout the incubation, bottles were weighed every two weeks to assess water content, and water losses were compensated. Following dosing, each bottle was shaken for 30 s to ensure uniform distribution of the solution throughout the soil.

Triplicate soil samples with each compound were immediately frozen at −20 °C upon application (day 0). Additional triplicate samples were removed from the incubator and frozen at 1, 2, 5, 12, 23, 40, 65, and 100 days post-application. All samples remained at −20 °C until compound extraction, which took place immediately after completing the degradation experiment. Short-term freezer storage at this temperature is not expected to influence compound concentrations (Fedorova et al., 2014).

Soil samples collected for degradation analysis were extracted and purified using the QuEChERS method (Anastassiades et al., 2003). Briefly, approximately 1 g of soil was weighed into an extraction vessel, 50 µL of the internal standard dissolved in acetonitrile was added, and left to evaporate solvent for 20 min (with the extraction vessel open). Subsequently, 10 mL of Milli-Q water was added. After manual mixing, 10 mL of acetonitrile were added, and extraction was performed on an automatic rotary shaker for 10 min. Subsequently, QuEChERS salts were added as follows: 1 g NaCl, 4 g MgSO_4_, 1 g Na_3_C_6_H_5_O_7_. 2H_2_O, and 0.5 g Na_2_C_6_H_5_O_7_. 1.5H_2_O. The sample was shaken vigorously for 1 min and then centrifuged (3 min, 10,000 rpm). An aliquot (1 mL) of the upper acetonitrile phase was transferred to a disposable glass tube and evaporated to dryness under a gentle stream of nitrogen. The residue was reconstituted in 2 mL of 50% methanol in Milli-Q water, filtered through a 0.22 µm cellulose membrane filter, and transferred to an amber glass vial for instrumental analysis.

All target analytes were determined by ultra-performance liquid chromatography coupled with tandem mass spectrometry (UPLC-MS/MS) described in previous section. However, the method was validated for samples extracted from a solid matrix, and therefore some parameters differed. The column temperature was set to 65 °C for all analytes, and injection volume of samples was 10 µL. As a mobile phase 5 mM ammonium acetate (A) and methanol (B) were used. Desolvation temperature was 550 °C. Detailed information on LC–MS/MS methods, including the LOQ range for the given analytes, is provided in the supplementary materials (Table S4).

Recoveries in % (Table S6) were calculated for each soil and compound as follows:

3 $$Recovery=100\frac{m {c}_{0}}{{V c}_{apl}}$$

where denominator is the initially applied compound load into the bottle (measured solute concentration c_apl_ (μg cm^−3^) in solution multiplied by its volume–V (6 or 4 cm^3^)) and numerator is the recovered compound amount at day 0 (solute concentration in soil–c_0_ (μg g^−1^) multiplied by soil mass–m (50 g)). The average recoveries in all 5 soils (%) and their standard deviations were: MET 105 ± 26, OA 105 ± 14, and ESA 122 ± 10. High recovery variability (up to 30%) is driven by measurement uncertainty within the soil matrix, specifically across the five soils with distinct properties.

Because mathematical models for simulating organic contaminant transport in soils typically assume first-order kinetics to describe compound dissipation (e.g., Beulke et al., 2020; Šimůnek et al., 2024), a first-order kinetic model was used to fit the experimental data (i.e., remaining concentrations at days 0, 1, 2, 5, 12, 23, 40, 65 and 100):

4 $$\frac{{c}_{t}}{{c}_{0}}={e}^{-{k}_{R}t}$$

where c_0_ (μg g^−1^) is the initial concentration, c_t_ (μg g^−1^) is the concentration in time, t (day) is time, and k_R_ (day^−1^) is the first-order rate constant. Logarithmic transformation was again applied to the data and Eq. (3), followed by the determination of unknown k_R_ coefficient via the least squares method (Table S6 and 2, Figure S2). Next, compound dissipation half-life DT_50_ (day) was calculated as follows (Table S6 and 2):

5 $${DT}_{50}=\frac{\mathrm{ln}2}{{k}_{R}}$$

During the batch experiments with each compound (MET, OA, and ESA), the concentrations of all three compounds and two additional metabolites (479M09 and 479M11) were always measured. Analyses of all five substances detected in experiments with individual metabolites OA and ESA demonstrated dissipation of both metabolites and negligible concentrations of other compounds (Figure S3). On the other hand, the experiment with the parent compound showed the dissipation of this substance and at the same time the formation and subsequent dissipation of all the metabolites examined (Figure S3 and 1). It is generally assumed that metazachlor transforms via the substitution of the chlorine atom by glutathione, followed by subsequent peptide cleavage and next via different pathways leading to various metabolites (APVMA, 2016). While the metabolic pathways for the metabolites OA, ESA, and 479M09 differ (APVMA, 2016), the metabolite 479M11 was considered to be transformed from the metabolite 479M09 (ECHA, 2011). On the other hand, the metabolic scheme for simulating the transformation of metazachlor using the FOCUS PELMO 5.5.3 model (BASF Se, 2020) assumed the parallel formation of all four metabolites. It should also be noted that the OA metabolite may alternatively be formed via a direct pathway (i.e., the reactive –CH₂–Cl group is directly converted into a –CO–COOH group), although the glutathione-dependent pathway remains the primary metabolic route in plants and soil (APVMA, 2016).

**Fig. 1:**
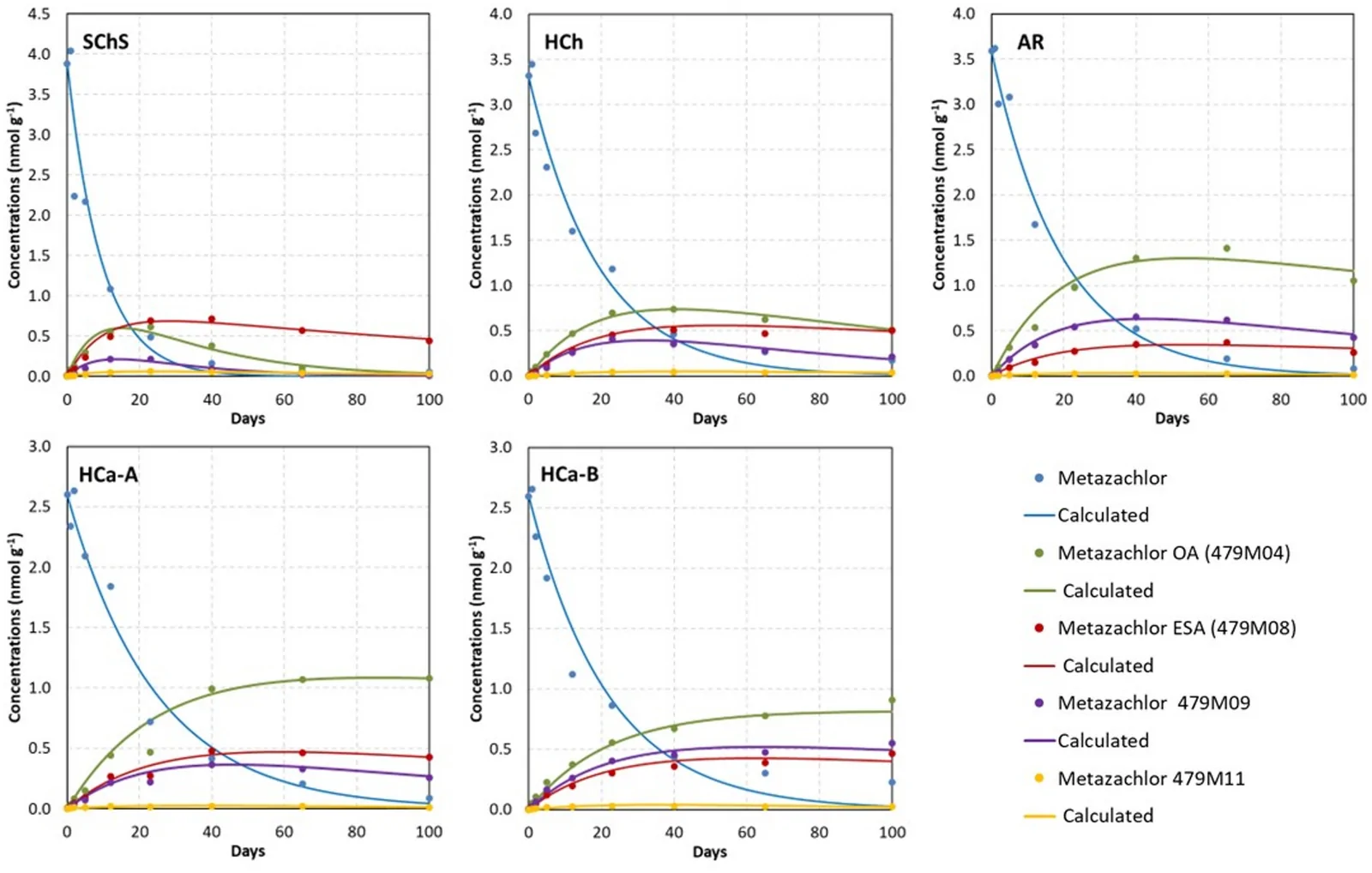
Measured and calculated concentrations of metazachlor and its metabolites during the degradation experiment in different soils: soils stagnic chernozem siltic (SChS), Haplic Chernozem (HCh), two Haplic Cambisol (HCa–A and HCa–B), and Arenic Regolol (AR)

In practical applications, such as simulating the behavior of compounds in the vadose zone, individual transformation steps cannot be accounted for. The problem is simplified to describe the transformation between the parent compound and its major metabolites. In the subsequent analyses, it was assumed that the metabolites do not form a chain, and the degradation of the parent compound, as well as the subsequent formation and dissipation of the individual metabolites, were calculated. Nevertheless, the approach described below can also be used to evaluate the degradation chain, i.e. calculate the possible transformation of metabolite 479M09 to 479M11, as implemented, for example, in the HYDRUS model for simulating water flow and contaminant transport in the vadose zone (Šimůnek et al., 2024) or in the HYDRUS module for calculating behavior within plant tissues, where the matrix is used to simulate the parallel, sequential, or even closed metabolization chains (Brunetti et al., 2019). Computing the direct transformation of the primary compound into its metabolite enables the evaluation of what proportion of the substance was transformed into each metabolite. First, using the molar masses of each compound, all concentrations were expressed in mmol per g of dry soil (mmol g^−1^). Next, the following approach was used to optimize the parameters of a first-order kinetic model describing the dissipation of the parent compound and the subsequent formation and simultaneous dissipation of each metabolite. The metazachlor dissipation profile (i.e., the fitted curves shown in the Fig. 1) was calculated with a narrow computation time step (t_j _− t_j–1_) using the modified original first-order kinetic equation as follows:

6 $$\frac{d{c}_{1}}{dt}=-{k}_{DR1}{c}_{1}$$

7 $${c}_{1,{t}_{j}}={c}_{1,{t}_{j-1}}-{k}_{DR1}{c}_{1,{t}_{j-1}}({{t}_{j}-t}_{j-1})$$

where c_1_ (mmol g^−1^) is metazachlor concentration and k_DR1_ (day^−1^) is the first-order rate constant, which was optimized by minimizing the following function, F_1_:

8 $${F}_{1}=\sum_{l=1}^{N}{\left({c}_{measured,1,{t}_{l}}-{c}_{calculated,1,{t}_{l}}\right)}^{2}$$

where N is the number of the measured concentrations over time.

Formation and dissipation of each metabolite was described as follows:

9 $$\frac{d{c}_{2}}{dt}={Kk}_{DR1}{c}_{1}-{Kk}_{DR2}{c}_{2}$$

where c_2_ (mmol g^−1^) is the metabolite concentration over time. The first term on the right-hand side of the equation represents the formation of a metabolite from the parent compound, and the second term represents the dissipation of that metabolite. K ( −) denotes the fraction of the parent compound that was transformed into a particular metabolite (i.e., yield). To keep information about the ratio between the values for the parent compound and the metabolite, the K value is also used in the second term describing dissipation of the metabolite and k_DR2_ (day^−1^) is the dissipation first-order rate constant for metabolite. The actual formation (k_FR2,actual_) and dissipation (k_DR2,actual_) first-order rates for the metabolite are:

10 $$k_{FR2,actual} = Kk_{DR1} \quad \quad k_{DR2,actual} = Kk_{DR2}$$

Assuming narrow computational time step (t_j_−t_j−1_), the c_2_ values over time t_j_ were calculated using the following equation:

11 $${c}_{2,{t}_{j}}={c}_{2,{t}_{j-1}}{+ Kk}_{DR1}{c}_{1,{t}_{j-1}}({t}_{j}{-t}_{j-1}){- Kk}_{DR2}{c}_{2,{t}_{j-1}}({t}_{j}{-t}_{j-1})$$

and the K and k_DR2_ values were optimized by minimizing the objective function, F_2_:

12 $${F}_{2}=\sum_{l=1}^{N}{\left({c}_{measured,2,{t}_{l}}-{c}_{calculated,2,{t}_{l}}\right)}^{2}$$

The actual first-order dissipation rates (k_DR1,actual_ = k_DR1_ and k_DR2,actual_) were next used to calculate dissipation half-lives, i.e., DT_50-1_ for the parent compound and DT_50-2_ for the metabolites, respectively, using Eq. 5. The ratios (R_KR_) between the actual formation (k_FR2,actual_) and dissipation (k_DR2,actual_) first-order rate constants for the metabolite were also computed.

13 $${R}_{KR}=\frac{{k}_{FR2,actual}}{{k}_{DR2,actual}}=\frac{{k}_{DR1}}{{k}_{DR2}}$$

If R_KR_=1, the formation and dissipation rates of metabolites are the same. If R_KR_<1, the dissipation rate is lower than the formation rate, and if R_KR_>1, the dissipation rate is higher than the formation rate. Results are in Table S7.

It should be noted that recently new tools have been developed for kinetic evaluation of a chemical degradation data (e.g., Ranke et al., 2018) using various functions, which could likely in few cases provide better fits of measured values. However, the resulting DT_50_ values could not be adopted in simulation models. In addition, Mantzos et al. (2017), who tested three different models, showed that degradation of MET followed the first-order kinetics.

It should also be noted that this method (Eqs. 6–13) was originally developed to calculate the dissipation rates of parent compounds and their metabolites based on data from non-target and suspect screening analyses, specifically, the signal intensities of parent and product compounds divided by the signal intensities of the corresponding internal standards (Nováková et al., 2024). In such a case, since the data did not represent the molar concentrations of the compounds but rather the analyte response corrected for sample processing losses and uncertainties, the K value did not merely indicate the fraction of the substance converted into the metabolite but also incorporated the conversion of analyte responses. Consequently, the K-value could take on any magnitude, including values greater than 1.

### *Statisticalanalysis*

To assess the goodness of fits using Eqs. 2, 4, 7, and 11 the coefficients of determination (R^2^) and* p*-values were calculated. Besides the means of fitted parameters standard errors where calculated, that can be used to compute lower and upper confidence limits. While these values for the optimized parameters of the logarithmically transformed linearized Eqs. 2 and 4 were evaluated using a standard functions in Excel software, in the case of Eqs. 7 and 11, the standard error were estimated from knowledge of the minimum of objective function, the number of observations, the number of unknown parameters to be fitted, and an inverse matrix describing the sensitivity of the model to changes in optimized parameters, i.e., derivatives calculated using finite differences when a small shift of the parameter has occurred (e.g., HYDRUS procedure for inverse modeling, Šimůnek et al., 2024). Next statistical analyses were performed using STAGRAPHICS Centurion XV Version 15.2.06. To investigate the relationships between the K_F_ (for n_avg_ value), k_R_, and DT_50_ values and various soil properties, Pearson correlation analysis was conducted (Tables S8 and S9), with significance determined by* p*-values. Standard measures of variability, and measures of shape, including the standardized skewness and standardized kurtosis, were assumed to determine whether the sample comes from a normal distribution. The same approach was also used to assess correlations between corresponding values K_F_ (for n_avg_ value), k_R_, and DT_50_ values and k_DR1-actual_, k_DR2-actual_, DT_50-1_, and DT_50-2_. Next, multiple linear regressions were used to create predictive models for estimating the K_F_, k_R_, and DT_50_ values based on soil properties (Table 3). Because the dataset contained comprehensive information for a limited number of soils (i.e., 8 and 5 for K_F_ and DT_50_, respectively), it was not possible to proceed via a backward elimination approach. That is, by initially including all soil properties and then successively eliminating data with the highest* p*-values. Therefore, a combined approach was adopted. Initially, various combinations of properties were selected and subsequently eliminated. Furthermore, a stepwise addition procedure was employed, in which the most effective properties were added one by one. 

## Results and discussion

### *Sorptionofcompoundsinsoils*

The initial Freundlich parameters, K_F_ and n, derived from the experimental data are summarized in Table S5. Table S5 and 1 represent the K_F_ values calculated using the average exponent n_avg_. Table 1 also provides comparisons with values reported in the literature for various soils. According to the coefficients of determination R^2^ in Table S5 and fits (Figure S1), using the average n value across all soils did not significantly worsen the fits of the data points. The K_F_ values for metazachlor varied between 0.235 and 2.94 cm^3/n^ μg^1−1/n^ g^−1^. The K_F_ values for metazachlor OA (0.096–2.84 cm^3/n^ μg^1−1/n^g^–1^) and ESA (0.083–0.307 cm^3/n^ μg^1−1/n^g^–1^) were similar and an order of magnitude lover than those for the parent compound.

**Table 1:** Freundlich sorption coefficients (K_F_), first-order dissipation rates (k_R_), and half-lives (DT_50_) obtained from the experiments for the individually applied compounds: MET–metazachlor, OA (M04)–metazachlor OA (479M04), and ESA (M08)–metazachlor ESA (479M08); and values published by EFSA (2017) and in PPDB (Lewis et al., 2016)

	K_F_ (cm^3/n^ μg^1−1/n^ g^−1^) for average n			k_R_ (day^−1^)			DT_50_ (day)		
n_avg_	1.19	1.21	1.13						
Soils	MET	OA (M04)	ESA (M08)	MET	OA (M04)	ESA (M08)	MET	OA (M04)	ESA (M08)
SChS	1.562	0.215	0.234	0.0519	0.0259	0.0094	13.4	26.8	74
HCh	1.304	0.156	0.147	0.0347	0.0074	0.0056	20	93	122.8
GP	1.030	0.121	0.083	NA	NA	NA	NA	NA	NA
HL	0.930	0.142	0.089	NA	NA	NA	NA	NA	NA
HCa-A	0.850	0.141	0.174	0.0407	0.0048	0.0059	17	144	116.9
HCa-B	1.618	0.172	0.184	0.0335	0.0044	0.008	20.7	157.7	86.3
DCa	2.941	0.284	0.307	NA	NA	NA	NA	NA	NA
AR	0.235	0.093	0.095	0.0402	0.0025	0.0011	17.2	273.9	618.1
Avg	1.309	0.165	0.164	0.0402	0.009	0.006	17.7	139.1	203.6
St.Dev	0.794	0.060	0.078	0.0073	0.0096	0.0032	2.9	91.2	232.6
Min	0.235	0.093	0.083	0.0335	0.0025	0.0011	13.4	26.8	74.0
Max	2.941	0.284	0.307	0.0519	0.0259	0.0094	20.7	273.9	618.1
EFSA/PPDB	0.48–4.4	0.014–1.57	0.037–0.39				5–25	19–507	51–362
	for n	for n	for n						
	0.83–1.43	0.65–1.59	0.89–1.23						

NA–not analyzed

However, it should be noted that some R^2^ values (according to EFSA (2017b) should be preferably above 0.975) and evaluated parameters’ deviations (Table S5), particularly for metabolites, exhibit increased prediction uncertainty. This was caused by the very low sorption of these substances in certain soils, and the consequent higher degree of uncertainty in evaluating the sorption isotherm points, which resulted in their significant scatter. To maintain a consistent approach, and to allow for comparison with our earlier data characterizing the sorption behavior of micropollutants (not only pesticides) in the soil environment, the same soil-to-aqueous-solution ratio (10 g of soil/20 cm^3^ of solution) for all three substances was used. EFSA (2017b), which established quality control and criteria regarding model fit and parameter reliability for batch adsorption experiments for pesticides, recommends that the distribution coefficient value (i.e., the s/c value) multiplied by the soil-to-aqueous-solution ratio be greater than 0.3. A different ratio, e.g., 1:1, would increase the fraction of sorbed substances and thus enhance the accuracy of the analysis. On the other hand, it should be noted that metabolite sorption is so low that any deviations from the determined values should not have a significant impact when assessing their mobility in the vadose zone. EFSA (2017b) also suggests that the range of initial concentrations should span two orders of magnitude (e.g., 0.1–10 μg cm^–3^). In several previous cases, the lowest concentration was too low and was therefore raised to 0.5. A sufficient concentration range is important for determining the Freundlich exponent. However, in the subsequent statistical analyses, the n values were averaged, and the resulting K_F_ parameters derived based on these mean n values were used. This reduced the importance of precisely determining the value of n for the overall assessment of sorption affinity in various soil units (e.g., Kodešová et al., 2011).

### *Degradationandtransformationofcompoundsinsoils*

The first-order degradation rates (k_R_) and half-lives (DT_50_) obtained from the experiments with single compound using Eqs. 3 and 4 (Figure S2) are presented in Table S6 and 1. The literature DT_50_ values for various soils are also included in Table 1. Resulting values show that in all cases the dissipation of the parent compound was much faster than dissipation of its two metabolites. For example, DT_50_ values (27–274 (OA) and 74–618 days (ESA)) were in some cases more than an order magnitude higher than those for the parent compound (13–21 days).

Characteristics describing transformation (i.e., degradation of metazachlor, and formation and transformation of its four metabolites) using Eqs. 6 and 10 (Fig. 1) are summarized in Table S7. The first-order degradation rates (k_DR1-actual_ and k_DR2-actual,_) and half-lives (DT_50-1_ and DT_50-2_) are also presented in Table 2. The values obtained for metazachlor using both approaches differ slightly, despite fitting the same data, because in the first case data were logarithmically transformed, while in the second case untransformed data were analyzed. In the first case low concentration values significantly influenced the curve fits, leading in some instances to an overestimation of the middle curve values (Figure S2). In the second case, low concentrations have a minor effect, yielding a better middle-curve fit, albeit with an underestimation of the terminal values, which may indicate the potential for long-term persistence of residual concentrations in the environment (Fig. 1). Results indicate that the parent compound dissipation (DT_50_ = 7–17 days) was much faster than dissipation of all four metabolites (18–492, 110–231, 15–111, and 39–87 days for OA (479M04), ESA (479M08), 479M09 and 479M11, respectively). Dissipation of OA and ESA metabolites was slower than that resulting from the degradation experiment when they were directly applied to soil. Interestingly in both cases (Table 1 and 2), dissipation of OA was much faster than ESA in SChS and HCh soil, while dissipation of ESA was much faster than OA in HCa–A and HCa–B soils. For E soil, in the first case, the OA dissipation was faster than the ESA dissipation, and in the second case, it was the other way around.

**Table 2:** Actual first-order dissipation rates (k_DR1-actual_ and k_DR2-actual_), corresponding half-lives (DT_50-1_ and DT_50-2_), and fractions of metabolites formed from the parent compound (K) obtained from the degradation experiment with metazachlor: MET–metazachlor, OA (M04)–metazachlor OA (479M04), ESA (M08)–metazachlor ESA (479M04), M09–metabolite 479M09, and M11–metabolite 479M11; and values published by EFSA (2017) and in PPDB (Lewis et al., 2016)

	First-order dissipation rates (day^−1^)					Half-lives (day)					Fractions of formed metabolites				
	K_DR1-actual_	K_DR2-actual_				DT_50-1_	DT_50-2_				K				
Soils	MET	OA (M04)	ESA (M08)	M09	M11	MET	OA (M04)	ESA (M08)	M09	M11	OA (M04)	ESA (M08)	M09	M11	Sum
SChS	0.1	0.0378	0.0063	0.045	0.012	6.9	18.3	110.0	15.4	57.8	0.27	0.21	0.1	0.02	0.6
HCh	0.051	0.00896	0.0042	0.016	0.008	13.6	77.4	165.0	43.3	86.6	0.32	0.21	0.2	0.02	0.75
HCa–A	0.04	0.00141	0.0048	0.0099	0.018	17.3	491.6	144.4	70.0	38.5	0.47	0.24	0.22	0.02	0.95
HCa–B	0.045	0.00163	0.0032	0.00625	0.0143	15.4	424.7	216.6	110.9	48.5	0.34	0.2	0.25	0.026	0.816
AR	0.05	0.0022	0.003	0.00825	0.015	13.9	315.1	231.0	84.0	46.2	0.44	0.12	0.25	0.015	0.825
Avg	0.057	0.0104	0.0043	0.0171	0.0135	13.4	265.4	173.4	64.7	55.5	0.368	0.196	0.204	0.020	0.788
St.Dev	0.0243	0.0156	0.0013	0.0160	0.0037	3.9	209.4	50.3	36.8	18.7	0.084	0.045	0.062	0.0039	0.128
Min	0.04	0.00141	0.003	0.00625	0.008	6.9	18.3	110	15.4	38.5	0.27	0.12	0.1	0.015	0.6
Max	0.1	0.0378	0.0063	0.045	0.018	17.3	491.6	231	110.9	86.6	0.47	0.24	0.25	0.026	0.95
EFSA/PPDB						5 – 25	19 – 507	51 – 362	11 – 26	16 – 40	0.162	0.216	0.053	0.075	

Table 2 also shows fractions of metabolites formed from the parent compound (K). The K values show that generally the largest proportion of the parent compound was transformed to OA (0.27–0.47), less to ESA (0.12–0.24) and 479M09 (0.1–0.25), and the least (minimal fraction) to 479M11 (0.015–0.024). The sum of fractions of quantified metabolites was lowest in soil SChS (0.6) and further increased as follows HCh (0.75), HCa–B (0.816), AR (0.825), and HCa–A (0.95). The sum indicated the total yield of parallel-formed metabolites. However, assuming that metabolite 479M11 is derived from metabolite 479M09, only the first three values should be summed (though the contribution of 479M11 is negligible). The differences in the total yield were mainly due to different OA fractions. The low values were recorded when the optimization found lower DT_50_ values, i.e. faster transformation. This could lead to a lack of information about the actual concentration of compounds in the sample. It is also possible that, particularly in SChS and HCh soils, the compounds were transformed into other metabolites via different metabolic pathways as a result of biotic and abiotic factors specific to these soils. For example, the metabolite 479M12 (which was not analyzed) could have been formed not only through transformation from OA (BASF Se, 2020) but also from the parent compound via a metabolic pathway different from that of the OA, ESA, and 479M09 metabolites. Formation of non-extractable residues can be also assumed. For example Mamy et al. (2005) identified up to 40% non-extractable residues of metazachlor and its main metabolite 479M04. However, they used a different, less efficient extraction procedure (0.01 M solution of CaCl_2_ and methanol, respectively) for soils predominantly or moderately composed of CaCO_3_.

It should also be noted that some R^2^ values and deviations of the evaluated parameters (Tables S6 and S7), particularly regarding metabolites, exhibit increased prediction uncertainty. This uncertainty is the result of ability of the first-order kinetic equation to fit experimental points (as discussed above) as well as the high stability of metabolites (i.e., a small decrease in residual concentrations) and the slight variability of the measured values. The uncertainty could be partly reduced, for instance, by conducting measurements in greater detail, that is, using finer time intervals over a longer period. However, a longer incubation period can lead to significant changes in the biomass and activity of the microbial community due to the limited availability of labile carbon (Bradford et al., 2010; Kodešová et al., 2026a; Paul et al., 2001).

### Correlations between the K_F_, k_R_, and DT_50_ values for metazachlor and its metabolites and their relationships to soil properties

The correlation coefficients between the K_F_ values for different compounds (Table S8) indicated positive correlations between each other (e.g., 0.959, 0.939, and 0.867, for MET vs OA, OA vs ESA, and MET vs ESA, respectively). However, significance at 95% was not found between MET and ESA. These correlations indicated similar behavior in soils, confirmed by similar correlations with soil properties, particularly strong correlation with Cox content (0.705, 0.794, and 0.841, for MET, OA, and ESA respectively), which is typical for pesticide compounds. No relationships were observed between the K_F_ and k_R_ or DT_50_ values for metazachlor. Negative correlations were found between K_F_ and DT_50_ and positive correlations between K_F_ and k_R_ values for both metabolites. These correlations are surprising because it is usually assumed that sorbed substances would be less susceptible to degradation. It should be noted, however, that the sorption of these substances in soil is very weak. Furthermore, some K_F_ parameters were evaluated with a high degree of uncertainty. Therefore, these relationships do not have significant informative value. The correlation of k_R_ or DT_50_ values with soil properties was more likely random, for example, a significant positive correlation was found between k_R_ (ESA) and Cox, and negative correlations were obtained between k_R_ (OA) and ρ_s_, DT_50_ (OA) and clay content, etc.

### Correlations between the K_F_, k_R_, and DT_50_ values and corresponding values k_DR1-actual_, k_DR2-actual_, DT_50-1_, and DT_50-2_

The correlation coefficients between the K_F_ values and corresponding k_DR1-actual_, k_DR2-actual_, DT_50-1_, and DT_50-2_ values (Table S9) show similar patterns as correlations between K_F_, k_R_, and DT_50_ values (Table S8), However, no relationship was shown to be significant. Correlations between the corresponding values of k_R_ or DT_50_ and k_DR1-actual_ and k_DR2-actual_ or DT_50-1_ and DT_50-2_ were always positive, but only in one case (OA) were statistically significant.

Correlations between DT_50-1_ and DT_50-2_ for the parent and three metabolites (OA, ESA and 479M09) were positive, but not statistically significant. Accordingly, correlations between k_DR1-actual_ and k_DR2-actual_ for the same compounds were positive, but in four cases even statistically significant (MET vs OA, MET vs 479M09, OA vs 479M09, and ESA vs 479M09). On the other hand, correlations between values for the metabolite 479M11 with values for the parent compound and other three metabolites were negative. This may imply that, in the case of 479M11, dissipation was likely stimulated by different biotic and abiotic factors than in all other cases.

### Prediction of the K_F_, k_R_, and DT_50_

The equations for predicting the K_F_, k_R_, and DT_50_ values resulting from the multiple linear regressions are shown in Table 3. Analyses showed that the K_F_ values for all 3 compounds were positively related to Cox content as well as sand and silt content. Significance of sand and silt content was not as high as significance Cox content. Despite the R^2^ values being higher than those when considering Cox content only, two derived relationships (for metazachlor and metabolite ESA) were not statistically significant at the 95% level. For ESA, the alternative equation included a positive relationship to Cox and hydrolytic acidity (HA). Similar, but not statistically significant, relationships were found for OA. Positive correlations between K_F_ and Cox can be anticipated due to sorption via hydrophobic and other interactions with organic matter (Kodešová et al., 2023). However, as documented by Dechene et al. (2014) increase of organic matter via addition of biochar was irrelevant in the case of OA and ESA sorption in soil. The positive impact of HA on K_F_ was also found for organic micropollutants that were negatively charged as acidic metabolites OA and ESA (Kodešová et al., 2023). The positive effect of sand and silt is difficult to explain. In the case of both metabolites, this may be because their negatively charged molecules repel from the surface of negatively charged clay particles (which is confirmed by the negative relationship with clay content for ESA). Therefore, inversely, positive associations with increasing content of both fractions, can indicate that the effect of clay particles decreases. The positive correlation of the distribution coefficient with sand content was also observed for metazachlor by Parven et al. (2024a).

**Table 3:** Equations for predicting the Freundlich sorption coefficients (K_F_, cm^3/n^ μg^1−1/n^ g^−1^), half-lives (DT_50_, day), and first-order dissipation rates (k_R_, day^−1^) obtained from the experiments for the individually applied compounds using: oxidizable organic carbon content (Cox, %), silt and clay content (%), and hydrolytic acidity (HA, mmol^+^ 100 g^−1^)

Predicted value	Compound	Model	Equation	R^2^
K_F_	Metazachlor (MET)	A	K_F_MET_ = 0.236 + 0.694 Cox	49.7
		B	K_F_MET_ = − 6.15 + 0.837 Cox* + 0.0613 Sand + 0.0898 Silt	77.4
	Metazachlor OA	A	K_F_OA_ = 0.0745 + 0.0589 Cox*	63.1*
		B	K_F_OA_ = 0.0644 + 0.0569 Cox* + 0.000414 HA	67.6
		C	K_F_OA_ = − 0.434 + 0.0712 Cox** + 0.00502 Sand + 0.00703 Silt	88.3*
	Metazachlor ESA	A	K_F_ESA_ = 0.0376898 + 0.0818351*Cox**	70.7**
		B	K_F_ESA_ = 0.0145 + 0.0772 Cox** + 0.000954 HA	84.5**
		C	K_F_ESA_ = − 0.421 + 0.0953 Cox* + 0.00490 Sand + 0.00606 Silt	79.3
DT_50_	Metazachlor (MET)	A	DT_50_MET_ = 18.7** + 9.61 K_F_MET_*—6.91 Cox*	96.5*
		B	DT_50_MET_ = 19.6** + 10.6 K_F_MET_*—6.96 Cox*—0.0863 Clay	99.9*
	Metazachlor OA	A	DT_50_OA_ = − 199 + 9591 K_F_OA_ – 498 Cox—14.0 Clay*	99.9*
		B	DT_50_OA_ = 369**—55.0 Cox—6.20 Clay*	97.7*
	Metazachlor ESA	A	DT_50_ESA_ = 987—3585 K_F_ESA_ + 84.7 Cox—14.9 Clay	88.7
		B	DT_50_ESA_ = 753—104 Cox—16.9 Clay	83.2
k_R_	Metazachlor (MET)	A	k_R_MET_ = 0.0338**—0.0247 K_F_MET_* + 0.0177 Cox* + 0.000182 Clay	99.9*
		B	k_R_MET_ = 0.0357**—0.0227 K_F_MET_* + 0.0175 Cox*	97.6*
	Metazachlor OA	A	k_R_OA_ = 0.204—3.56 K_F_OA_ + 0.174 Cox + 0.00285 Clay	99.4
		B	k_R_OA_ = − 0.00634 + 0.00963 Cox—0.0000440 Clay	71.3
	Metazachlor ESA	A	k_R_ESA_ = − 0.00300 + 0.0361 K_F_ESA_ + 0.00116 Cox + 0.0000457 Clay	95.4
		B	k_R_ESA_ = 0.000140 + 0.00346 Cox*	90.2*
		C	k_R_ESA_ = − 0.000650 + 0.00307 Cox **+** 0.0000658 Clay	92.3

^**^ p < 0.01, * p < 0.05

The DT_50_ values for MET and OA were positively related to the K_F_ values and negatively related to Cox and clay content. The DT_50_ values for ESA and alternatively OA were negatively related to Cox and clay content. The k_R_ values for MET and OA were negatively related to the K_F_ values and positively related to Cox and clay content. The DT_50_ values for ESA are negatively related to Cox content. Mostly inverse relationships were obtained for k_R_ values and soil properties. Negative correlations between DT_50_ and K_F_ can be explained by the assumption that sorbed substances are less susceptible to degradation. Negative correlations between DT_50_ and Cox and clay content can be attributed to better conditions for microbial abundance and activity, i.e., available nutrients and better aeration (e.g., Koubová et al., 2026a). The positive correlations between the k_R_ values for MET and total organic carbon and clay content, i.e., negative between the DT50 values and total organic carbon and clay content, was also observed by Parven et al. (2024b)

## Novelty of findings in the context of present knowledge

A comparison of the resulting K_F_ values (Tables S5 and 1) for metazachlor and its two metabolites OA and ESA with literature data (Table 1) reveals that our results fall within the published range (e.g., EFSA, 2017a; Lewis et al., 2016). In the case of the OA metabolite, however, they are rather closer to the lower limit of the range of literature values. Both metabolites show in our study an order of magnitude lower K_F_ values (i.e., an order of magnitude higher mobility) than those of parent compound. It should be noted that the mobility of compounds in soils is also influenced by the current concentration of the compounds, and thus by the value of n, as well as by other soil properties such as soil water content and soil bulk density. While the n coefficients evaluated in this study for MET and its metabolites OA and ESA are similar, they are highly variable in the available database, which inevitably influenced the reported K_F_ values.

Half-lives (Tables S6 and 1) for metazachlor and its two metabolites OA and ESA are consistent with previously reported data (Table 1; e.g., EFSA, 2017a). However, the lower limit of the value range is higher in all cases and in loamy sand (AR) the half-life of ESA is almost 2 times higher than the upper maximum of the literature values.

The resulting half-life values for four metabolites of metazachlor formed and dissipated during the metazachlor degradation experiment (Table S7 and 2) show even greater differences compared to literature data. While in SChS and HCh, the DT_50-2_ values for OA were lower than the DT_50_ values (Table 1), the values in the other soils and particularly in both Cambisols were much higher (i.e., 3.4 and 2.7 times in HCa–A and HCa–B, respectively). Values of DT_50-2_ for ESA (Table 2) were, except AR, always higher than the DT_50_ values (Table 1), i.e., 1.5, 1.3, 1.2, and 2.5 times in SChS, HCh, Hca–A, and Hca–B, respectively. The resulting values are therefore closer to the upper limit of literature values or even exceed it. Similarly, the half-lives (DT_50-2_) of the remaining two metabolites 479M09 and 479M11 partially agree with published data, but some values are several times higher than the published maximum values, suggesting a higher stability of these metabolites in the environment than anticipated. However, the stability of both metabolites 479M09 and 479M11 is generally lower than that of OA and ESA metabolites. Interestingly, in soils SChS and HCh (developed on marlite and loess, respectively), DT_50_ and DT_50-2_ for ESA were higher than those for OA, and in Cambisols (HCa–A and HCa–B) developed on paragneiss it was the opposite, i.e., DT_50_ and DT_50-2_ for ESA were lower than those for OA. Value of DT_50-2_ for ESA was also lower than that for OA. Similarly for other two metabolites, while DT_50-2_ for 479M11 were higher than DT_50-2_ for M09 in soils SChS and HCh, in remaining three soils DT_50-2_ for 479M11 were lower than DT_50-2_ for M09. This could indicate a similar sensitivity of these metabolites OA and 479M09 or ESA and 479M11 to soil microbial conditions.

Very important and even alarming is the result showing the fraction of metabolites (K) that can be formed during the degradation of the parent compound. The published K value of 0.216 (Lewis et al., 2016) for ESA approximately agrees with our findings. However, the published K value (0.162) for OA is much lower than maximal (2.9 times) and even minimal (1.7 times) K values obtained in this study (Table 2). Similarly, the published K value (0.053) for 479M09 is much lower than found maximal (4.7 times) and even minimal (1.9 times) K values. Contrary to PPDB (Lewis et al., 2016) assumption that 479M09 is a minor component during the metazachlor transformation, our study found it to be nearly comparable to the ESA fraction, however, it exhibits a faster degradation rate. On the other hand, fraction of 479M11 was similar as reported. While according to PPDB (Lewis et al., 2016), the toxicity of the OA and ESA metabolites is not relevant, both 479M09 and 479M11 metabolites are considered to be potentially carcinogenic. In addition, both metabolites are also very mobile in soil environment. The average K_F_ coefficients for three soils published by EFSA (2017a) are 0.112 cm^3/n^ μg^1−1/n^ g^−1^ for n = 1.11 and 0.384 cm^3/n^ μg^1−1/n^ g^−1^ for n = 1.16 for 479M09 and 479M11, respectively. Our results thus show the possible threats arising from the occurrence of the metabolite 479M09, which decomposes faster in soil than OA and ESA, but may be much more stable in the vadose zone and groundwater.

Correlation and multiple regression showed that K_F_ coefficients are not dependent only on the Cox (i.e., organic matter) content and thus indicated a limited ability to predict K_F_ values using Koc values (e.g., Kodešová et al., 2011). This was particularly expected for the metabolites OA and ESA, as the sorption of negatively charged organic molecules is affected by several other mechanisms in addition to hydrogen bonding, π–π and hydrophobic interaction with organic matter, including cation bridging and even repulsion from the negatively charged surface of soil components (e.g., Kodešová et al., 2023, 2026b). Interestingly, however, a significant correlation between K_F_ and Cox was not found even for the parent compound, and this compound showed similar behavior as its metabolites. The dissipation half-lives were negatively dependent on the contents of Cox and clay, which probably indicated the influence of soil conditions (i.e. available nutrient and likely also soil aeration) on the composition and activity of the microbial community (Kodešová et al, 2020, 2026a; Koubová et al., 2026). In the case of metazachlor and OA metabolite, half-lives also positively correlated with K_F_, which may be related to the reduced availability of the sorbed compound for microbial degradation (e.g., Kodešová et al, 2020; Menacherry et al., 2023). However, it should be noted that the substances were only very slightly sorbed in soils. The equation derived for predicting the sorption and dissipation of studied compounds can be used to estimate their behavior in the soil environment, e.g. for predicting the distribution of properties within agricultural areas (as shown, for example, by Kodešová et al., 2011, 2023) and subsequently assessing the vulnerability of groundwater to contamination by the specific substances and defining particularly vulnerable areas.

## Conclusions

The K_F_ coefficient of metazachlor was an order of magnitude higher than those found for the metabolites OA and ESA. Sorption of all compounds in soils was primarily driven by the soil organic carbon content, but it was also affected by other properties such as texture and hydrolytic acidity (metabolites). The dissipation rates of the metabolites OA and ESA were an order of magnitude lower than that of the parent compound. Multiple linear regressions revealed the positive effect of the soil organic carbon and clay contentment and in some cases the negative effect of the sorption coefficient. Metazachlor and particularly its metabolites OA and ESA were less sorbed and were more stable than can be anticipated based on published and recommended values. Even higher metabolite stability was found during the transformation of the parent compound into its 4 metabolites: OA, ESA, 479M09 and 479M11. The dissipation rates of 479M09 and 479M11 were generally higher than those of OA and ESA but considerably lower than the parent compound dissipation rates. The sum of fractions of all four metabolites derived from the parent compound ranged between 60 and 95%. Fraction of OA and particularly potentially carcinogenic 479M09 metabolites formed during these experiments were much higher than has been reported in available databases. This indicates possible environmental risks associated with the occurrence of metabolite 479M09, although it degrades faster in soil than OA and ESA, it may be much more stable if it leaches into groundwater, where biodegradation is limited. Presented values describing sorption, formation and dissipation and equations for predicting the behavior of compounds in the soil environment provide essential information that will help improve the assessment of the possible spread of the studied substances in the soil and aquatic environment and the associated risks.

## Supplementary Information

Below is the link to the electronic supplementary material.

Supplementary file1 (PDF 1695 KB)

## Data Availability

All data supporting the findings of this study are available within the paper and its Supplementary Information. Data will be made available on request.
