# Supplementary material for: Persistence and mobility of metazachlor and its metabolites in soils: Why transformation products pose a greater groundwater risk
Source: Environ Geochem Health. 2026 Sep 2;48(14):567. doi: 10.1007/s10653-026-03452-w (PMC13538165; doi:10.1007/s10653-026-03452-w)
Supplement: Supplementary file 1 [file 10653_2026_3452_MOESM1_ESM.pdf]

Persistence and mobility of metazachlor and its metabolites in soils: Why transformation products pose a greater groundwater risk

Radka Kodešová[1*], Daniela Tomešová[2], Martin Kočárek[1], Miroslav Fér[1], Aleš Klement[1], Antonín Nikodem[1], Jindřich Zelinka[1], Vít Kodeš[3]

[1] Czech University of Life Sciences Prague, Faculty of Agrobiology, Food and Natural Resources, Dept. of Soil Science and Soil Protection, Kamýcká 129, CZ-16500 Prague 6, Czech Republic

[2] ALS Czech Republic, Project Department, Na Harfě 336/9, CZ-19000, Prague 9, Czech Republic

[3] Czech Hydrometeorological Institute, Na Šabatce 2050/17, CZ-14306 Praha 4, Czech Republic

*Corresponding author: Email: kodesova@af.czu.cz

Supplementary materials:

9 tables S1 – S9 and 3 figures S1 – S3

Table S1. Characteristics of metazachlor and its metabolites (http://sitem.herts.ac.uk/aeru/ppdb/en/index.htm).

Compound	CAS number	MW (g mol^{-1})	Water solubility (mg L^{-1})	pKa	log Kow	Strucural formula [d]
**Metazachlor**	67129-08-2	277.75	450	16.65 (acidic)<br>1.84 (basic) [e]	2.49	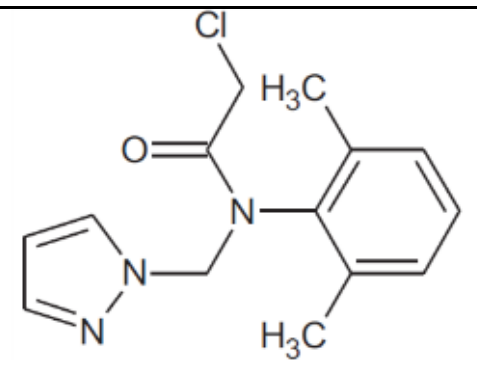
**Metazachlor oxalic acid (OA), 479M04**	1231244-60-2	273.29	198000 [b]	3,3 [b]	<2 [b]	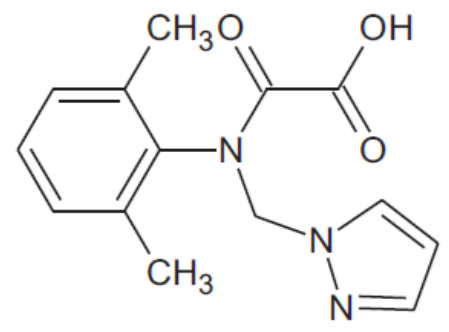
**Metazachlor ethane sulfonic acid ESA, 479M08**	172960-62-2	323.37	216000 [b]	1,6 [b]	<2 [b]	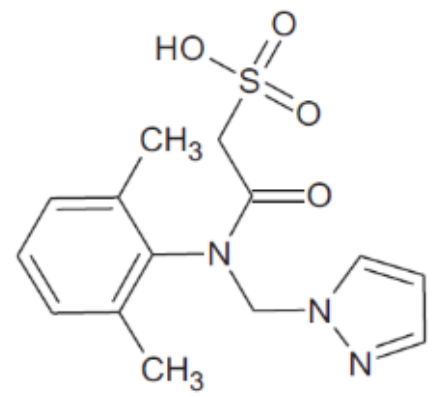
**Metazachlor M09, 479M09**	1246215-97-3	349.4[a]	soluble	NA [f]	1 [a]	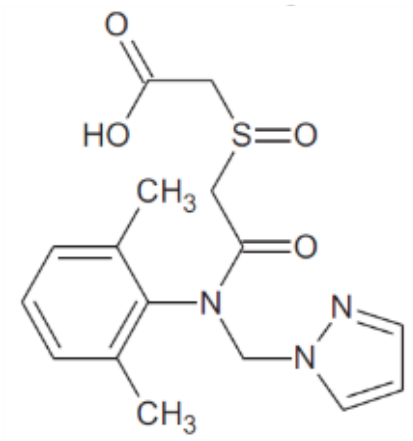
**Metazachlor M11, 479M11**	1242182-77-9	305.4[a]	soluble	1.53 [d]	1.3 [a]	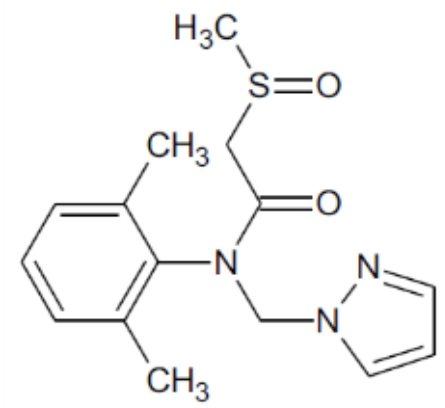

[a] – https://pubchem.ncbi.nlm.nih.gov, [b] – Dechene et al. (2014), [c] – EFSA (2017), [d] – https://www.chemicalbook.com, [e] – https://hmdb.ca/metabolites/HMDB0254508, [f] – Not available (anion in expected pH range)

Table S2. Soils Stagnic Chernozem Siltic on marlite (SChS), Haplic Chernozem on loess (HCh), Greyic Phaeozem on loess (GP), Haplic Luvisol on loess (HL), two Haplic Cambisol on paragneiss (HCa-A and HCa-B), Dystric Cambisol on orthogneiss (DCa), and Arenic Regolol (AR) on sand, and their properties: pH_{H2O}, pH_{KCl}, $pH_{CaCl2,}$, organic carbon content (Cox), soil salinity (electrical conductivity – EC), particle density (ρ_s), fraction of clay, silt and sand, cation exchange capacity (CEC), hydrolytic acidity (HA), base cation saturation (BCS), sorption complex saturation (SCS.

**Soil type**	**Texture**	**pH_{H2O}**	**pH_{KCl}**	**pH_{CaCl2}**	**Cox**	**$CaCO_3$**	**EC**	**ρ_s**	**Sand**	**Silt**	**Clay**	**CEC**	**HA**	**BCS**	**SCS**
			**(–)**		**(%)**		**($\mu S\ cm^{-1}$)**	**($g\ cm^{-3}$)**		**(%)**			**($mmol^+\ 100\ g^{-1}$)**		**(%)**
**SChS**	Loam	8.5	7.6	7.5	2.93	23.8	226.0	2.45	26.4	46.7	26.9	244.1	3.7	240.4	98.5
**HCh**	Silty clay loam	7.9	7.3	7.2	1.55	4.5	201.0	2.58	11.5	57.8	30.7	202.3	5.2	197.1	97.4
**GP**	Silty clay loam	7.4	6.3	6.4	0.97	0.6	104.5	2.57	10.0	62.6	27.4	151.2	10.4	140.8	93.1
**HL**	Silty loam	6.8	6.1	5.8	0.72	0.1	59.6	2.57	5.1	74.3	20.7	123.5	16.2	107.2	86.9
**HCa-A**	Loam	4.7	3.6	4.1	1.34	0	152.0	2.57	31.8	43.7	24.6	138.8	83.3	55.5	40.0
**HCa-B**	Loam	5.8	4.3	5.0	2.05	0	47.0	2.55	44.6	36.1	19.4	129.4	54.2	75.2	58.1
**DCa**	Silty loam	5.8	4.7	5.1	2.20	0	110.6	2.53	16.8	62.9	20.4	175.3	64.6	110.6	63.1
**AR**	Loamy sand	6.2	4.9	5.2	0.60	0	38.8	2.59	81.6	9.9	8.5	45.2	17.2	28.0	62.0
**Avg.**		6.6	5.6	5.8	1.54		117.4	2.55	28.5	49.2	22.3	151.2	31.9	119.4	74.9
**St.Dev.**		1.3	1.4	1.2	0.81		70.4	0.05	25.1	20.1	6.9	59.1	30.8	71.5	21.9
**Max.**		4.7	3.6	4.1	0.60		38.8	2.45	5.1	9.9	8.5	45.2	3.7	28.0	40.0
**Min.**		8.5	7.6	7.5	2.93		226.0	2.59	81.6	74.3	30.7	244.1	83.3	240.4	98.5

Table S3. Correlation coefficients describing relationship between soil properties: pH_{H2O}, pH_{CaCl2}, pH_{KCl}, salinity, EC (µS cm^{-1}), cation exchange capacity, CEC ($mmol^+$ 100 g^{-1}), hydrolytic acidity, HA ($mmol^+$ 100 g^{-1}), base cation saturation, BCS ($mmol^+$ 100 g^{-1}), sorption complex saturation, SCS (%), organic carbon content, Cox (%),particle density, ρ_s (g cm^{-3}), and sand, silt and clay contents (%).

	**pH_{H2O}**	**pH_{CaCl2}**	**pH_{KCl}**	**EC**	**CEC**	**HA**	**BCS**	**SCS**	**Cox**	**ρ_s**	**Sand**	**Silt**
**pH_{CaCl2}**	0.993***											
**pH_{KCl}**	0.986***	0.987***										
**EC**	0.519	0.596	0.569									
**CEC**	0.581	0.650	0.610	0.867**								
**HA**	-0.903**	-0.862**	-0.885**	-0.196	-0.183							
**BCS**	0.870**	0.909**	0.885**	0.801*	0.906**	-0.582						
**SCS**	0.966***	0.957***	0.975***	0.426	0.541	-0.893**	0.832*					
**Cox**	0.262	0.313	0.219	0.587	0.777*	0.128	0.588	0.126				
**ρ_s**	-0.430	-0.435	-0.386	-0.547	-0.716*	0.110	-0.639	-0.300	-0.874**			
**Sand**	-0.348	-0.392	-0.441	-0.418	-0.647	0.119	-0.586	-0.516	-0.166	0.163		
**Silt**	0.271	0.302	0.368	0.258	0.525	-0.090	0.473	0.468	0.078	-0.108	-0.978***	
**Clay**	0.472	0.547	0.531	0.774*	0.831*	-0.166	0.758*	0.516	0.380	-0.282	-0.797**	0.655

*** $p<0.001$, ** $p<0.01$, * $p<0.05$

Table S4. Data on LC-MS/MS method validation for selected pesticides and metabolites. Precursor and product ions, cone voltage (CV), retention times (RT), limits of quantification (LOQ), recoveries (REC) and relative standard deviations (RSD) are shown for both methods.

			Sorption				Degradation / dissipation			
**Compounds**	**Precursor ion (Product ions) (m/z)**	**CV (V)**	**RT (min)**	**LOQ (μg L^{-1})**	**REC (%)**	**RSD (%)**	**RT (min)**	**LOQ (μg L^{-1})**	**REC (%)**	**RSD (%)**
Metazachlor	278 (134/210)	30	7.7	0.004	111	4.6	5.9	0.0009	110	12.2
Metabolite 479M04 – OA	274 (134/162)	25	3.8	0.007	97	11.4	3.1	0.0008	105	9.6
Metabolite 479M08 – ESA	324 (134/69)	25	4.1	0.009	101	15.8	3.4	0.0012	107	6.8
Metazachlor metabolite 479M09	350.1 (134/282)	30	NA	NA	NA	NA	3.2	0.0005	103	11.8
Metazachlor metabolite 479M11	306.1 (134/238)	30	NA	NA	NA	NA	4.2	0.0005	114	7.7

NA – not analyzed

Table S5. Freundlich sorption coefficients, K_F ($cm^{3/n}$ $\mu g^{1-1/n}$ g^{-1}), n values (-), and coefficients of determination (R^2) indicating goodness of fit: Stagnic Chernozem Siltic on marlite (SChS), Haplic Chernozem on loess (HCh), Greyic Phaeozem on loess (GP), Haplic Luvisol on loess (HL), two Haplic Cambisol on paragneiss (HCa-A and HCa-B), Dystric Cambisol on orthogneiss (DCa), and Arenic Regolols on sand (AR).

Soil	Metazachlor			Metazachlor OA			Metazachlor ESA			Metazachlor n=1.19		Metazachlor OA n=1.21		Metazachlor ESA n=1.13	
	K_F	n	R^2	K_F	n	R^2	K_F	n	R^2	K_F	R^2	K_F	R^2	K_F	R^2
**SChS**	1.913	1.310	0.930***	0.119	0.9239	0.9207***	0.2615	1.151	0.705***	1.562	0.916***	0.215	0.926***	0.2345	0.702***
	± 0.077	± 0.054		± 0.025	± 0.1027		± 0.0276	± 0.112		± 0.090		± 0.015		± 0.0237	
**HCh**	1.394	1.242	0.965***	0.085	0.9948	0.7917***	0.1353	1.067	0.824***	1.304	0.964***	0.156	0.783***	0.1475	0.827***
	± 0.041	± 0.037		± 0.029	± 0.1770		± 0.0148	± 0.102		± 0.043		± 0.016		± 0.0108	
**GP**	0.465	0.772	0.975***	0.047	0.9554	0.4756*	0.0881	1.222	0.495**	1.030	0.933***	0.121	0.480**	0.0830	0.492*
	± 0.027	± 0.025		± 0.030	± 0.2274		± 0.0145	± 0.173		± 0.079		± 0.025		± 0.0148	
**HL**	1.024	1.263	0.962***	0.119	1.1821	0.4308	0.1202	1.557	0.498	0.930	0.959***	0.142	0.433	0.0893	0.491*
	± 0.034	± 0.043		± 0.065	± 0.2774		± 0.0307	± 0.367		± 0.033		± 0.032		± 0.0146	
**HCa-A**	0.881	1.170	0.9582***	0.174	1.5721	0.3271	0.1681	1.001	0.875***	0.850	0.960***	0.141	0.288	0.1743	0.890***
	± 0.035	± 0.045		± 0.061	± 0.4046		± 0.0206	± 0.099		± 0.030		± 0.036		± 0.0098	
**HCa-B**	1.707	1.198	0.9642***	0.214	1.4456	0.4349**	0.1913	1.078	0.807***	1.618	0.964***	0.172	0.414*	0.1842	0.813***
	± 0.059	± 0.041		± 0.056	± 0.2763		± 0.0235	± 0.367		± 0.055		± 0.033		± 0.0146	
**DCa**	2.929	1.135	0.9620***	0.254	1.1216	0.7720***	0.2627	0.987	0.812***	2.941	0.965***	0.284	0.824***	0.2846	0.824***
	± 0.098	± 0.035		± 0.040	± 0.1273		± 0.0277	± 0.096		± 0.097		± 0.031		± 0.0224	
**AR**	0.329	1.433	0.8160***	0.116	1.5274	0.9155***	0.0724	0.927	0.361	0.235	0.783***	0.093	0.925***	0.0754	0.411*
	± 0.029	± 0.136		± 0.029	± 0.3030		± 0.0312	± 0.102		± 0.026		± 0.006		± 0.0118	
**Avg.**	1.330	1.190		0.141	1.215		0.162	1.124		1.309		0.165		0.159	0.681
**St.Dev.**	0.853	0.193		0.068	0.264		0.073	0.198		0.794		0.060		0.076	0.188
**Min**	0.329	0.772		0.047	0.924		0.072	0.927		0.235		0.093		0.075	0.411
**Max**	2.929	1.433		0.254	1.572		0.263	1.557		2.941		0.284		0.285	0.890

*** p<0.001, ** p<0.01, * p<0.05

Table S6. First-order dissipation rates (k_R), half-lives (DT_{50}), coefficients of determination (R^2) and recoveries obtained from the experiments for the individually applied compound: MET – metazachlor, OA (M04) – metazachlor OA (479M04), and ESA (M08) – metazachlor ESA (479M08): Stagnic Chernozem Siltic on marlite (SChS), Haplic Chernozem on loess (HCh), two Haplic Cambisol on paragneiss (HCa-A and HCa-B), and Arenic Regolols on sand (AR).

**Soil**	**k_R (day^{-1})**	**R^2**	**k_R (day^{-1})**	**R^2**	**k_R (day^{-1})**	**R^2**	**DT_{50} (day)**			**Recovery (%)**		
	**MET**		**OA (M04)**		**ESA (M08)**		**MET**	**OA (M04)**	**ESA (M08)**	**MET**	**OA (M04)**	**ESA (M08)**
**SChS**	0.0519	0.885	0.0259	0.882	0.0094	0.95	13.4	26.8	74.0	127	128	121
	±0.005	***	±0.002	***	±0.001	***	±1.2	±2.1	±5.4			
**HCh**	0.0347	0.952	0.0074	0.935	0.0056	0.897	20.0	93	122.8	108	108	129
	±0.003	***	±0.0005	***	±0.0005	***	±1.4	±5.4	±9.4			
**HCa-A**	0.0407	0.958	0.0048	0.851	0.0059	0.938	17.0	144.0	116.9	73	98	122
	±0.003	***	±0.001	**	±0.001	***	±1.01	±23.3	±11.1			
**HCa-B**	0.0335	0.938	0.0044	0.83	0.008	0.84*	20.7	157.7	86.3	85	103	106
	±0.004	***	±0.001	**	±0.001	***	±2.2	±16.5	±11.4			
**AR**	0.0402	0.971	0.0025	0.823	0.0011	0.27	17.2	273.9	618.1	134	90	130
	±0.002	***	±0.0005	**	±0.001		±0.7	±43.8	±240.4			
**Avg.**	0.0402		0.009		0.006		17.7	139.1	203.6	105	105	122
**St.Dev.**	0.0073		0.0096		0.0032		2.9	91.2	232.6	26	14	10
**Min**	0.0335		0.0025		0.0011		13.4	26.8	74	73	90	130
**Max**	0.0519		0.0259		0.0094		20.7	273.9	618.1	136	128	103

*** p<0.001, ** p<0.01, * p<0.05

Table S7. Values obtained from fitting measured concentrations of the parent compound and its metabolites during the degradation experiments with metazachlor: fraction of the parent compound that was transformed into a particular metabolite (K), first-order dissipation rate for metazachlor and its metabolites (k_{DR1} and k_{DR2}), respectively, actual first-order dissipation rate for metazachlor and its metabolites ($k_{DR1-actual}$ and $k_{DR2-actual}$), respectively, first-order formation rate constant for metabolites k_{FR2}, objective function F (F_1 or F_2), coefficient of determination (R^2), and half-lives (DT_{50-1} and DT_{50-2}) calculated from $k_{DR1-actual}$ and $k_{DR2-actual}$, respectively. The p-values were mostly lower than 0.001 except for metabolite M11 (0.0012) in HCa-A. Given that all p-values were below 0.05, the fit of the experimental data demonstrated a statistically significant match at a 95.0% confidence level (even higher).

	**Soil**	**Metazachlor**	**Metazachlor OA (479M04)**	**Metazachlor ESA (479M08)**	**Metazachlor 479M09**	**Metazachlor 479M11**	**Sum K**
K (−)	SChS		0.27±0.09	0.21±0.08	0.10±0.08	0.020±0.019	0.60
	HCh		0.32±0.13	0.21±0.048	0.20±0.08	0.020±0.018	0.75
	HCa-A		0.47±0.12	0.24±0.11	0.22±0.07	0.020±0.021	0.95
	HCa-B		0.34±0.14	0.20±0.041	0.25±0.06	0.026±0.022	0.816
	AR		0.44±0.12	0.12±0.07	0.25±0.07	0.015±0.016	0.825
k_{DR1} for metazachlor	SChS	0.10±0.06	0.14±0.003	0.03±0.012	0.45±0.001	0.60±0.17	
k_{DR2} for metabolites	HCh	0.051±0.017	0.028±0.013	0.02±0.008	0.08±0.007	0.40±0.12	
Fitted values	HCa-A	0.04±0.008	0.003±0.00008	0.02±0.006	0.045±0.0007	0.90±0.24	
(days^{-1})	HCa-B	0.045±0.012	0.0048±0.0021	0.016±0.0037	0.025±0.003	0.55±0.005	
	AR	0.05±0.008	0.005±0.0002	0.025±0.0006	0.033±0.0002	1.0±0.0002	
F ((mmol g^{-1})^2)	SChS	1.231	0.024	0.021	0.005	0.00007	
	HCh	0.341	0.004	0.011	0.008	0.00004	
	HCa-A	0.246	0.077	0.010	0.009	0.00018	
	HCa-B	0.253	0.005	0.005	0.001	0.00029	
	AR	0.299	0.045	0.003	0.006	0.00008	
R^2	SChS	0.942	0.949	0.976	0.931	0.984	
	HCh	0.976	0.994	0.985	0.967	0.981	
	HCa-A	0.974	0.961	0.968	0.959	0.845	
	HCa-B	0.967	0.977	0.966	0.946	0.903	
	AR	0.983	0.972	0.968	0.991	0.974	
$k_{DR1-actual} = k_{DR1}$	SChS	0.10	0.0378	0.0063	0.045	0.012	
$k_{DR2-actual} = K*k_{DR2}$	HCh	0.051	0.00896	0.0042	0.016	0.008	
Actual dissipation rates	HCa-A	0.04	0.00141	0.0048	0.0099	0.018	
(days^{-1})	HCa-B	0.045	0.00163	0.0032	0.00625	0.0143	
	AR	0.05	0.0022	0.003	0.00825	0.015	
K_{FR2} (days^{-1}) = K* k_{DR1}	SChS		0.027	0.021	0.01	0.002	
Actual formation rates	HCh		0.01632	0.0107	0.0102	0.00102	
metabolites	HCa-A		0.0188	0.0096	0.0088	0.0008	
(days^{-1})	HCa-B		0.0153	0.009	0.0114	0.00117	
	AR		0.022	0.006	0.0125	0.00075	
$R_{KR} = k_{FR2-actual} / k_{DR2-actual}$	SChS		0.71	3.33	0.22	0.17	
	HCh		1.82	2.55	0.64	0.13	
	HCa-A		13.3	2	0.89	0.044	
	HCa-B		9.4	2.81	1.8	0.082	
	AR		10	2	1.52	0.05	
DT_{50-1}	SChS	6.9	18.3	110.0	15.4	57.8	
DT_{50-2}	HCh	13.6	77.4	165.0	43.3	86.6	
(days)	HCa-A	17.3	491.6	144.4	70.0	38.5	
	HCa-B	15.4	424.7	216.6	110.9	48.5	
	AR	13.9	315.1	231.0	84.0	46.2	

Table S8. Correlation coefficients describing relationships between the Freundlich sorption coefficients, K_F ($cm^{3/n}$ $\mu g^{1-1/n}$ g^{-1}), half-lives, DT_{50} (days), and first - order dissipation rates, k_R (days-1), for the studied compounds metazachlor (MET) and its metabolites OA and ESA, and between these values and soil properties: pH_{H2O}, pH_{CaCl2}, pH_{KCl}, salinity, EC (μS cm^{-1}), cation exchange capacity, CEC ($mmol^+$ 100 g^{-1}), hydrolytic acidity, HA ($mmol^+$ 100 g^{-1}), base cation saturation, BCS ($mmol^+$ 100 g^{-1}), sorption complex saturation, SCS (%), organic carbon content, Cox (%), particle density, ρ_s (g cm^{-3}), and sand, silt and clay contents (%).

		**K_F** (8 soils)			**K_F** (5 soils)			**DT_{50}** (5 soils)			**k_R** (5 soils)		
		**MET**	**OA**	**ESA**	**MET**	**OA**	**ESA**	**MET**	**OA**	**ESA**	**MET**	**OA**	**ESA**
**K_F** (8 or 5 soils)	**MET**	1			1								
	**OA**	0.959***	1		0.913*	1							
	**ESA**	0.867	0.939***	1	0.817	0.953*	1						
**DT_{50}** (5 soils)	**MET**				0.097			1					
	**OA**					-0.917*		0.343	1				
	**ESA**						-0.832	-0.047	0.843	1			
**k_R** (5 soils)	**MET**				0.0410			-0.989**			1		
	**OA**					0.816			-0.801		0.840	1	
	**ESA**						0.967**			-0.909*	0.311	0.670	1
**Soil properties**	**pH_{H2O}**	-0.011	0.032	-0.132	0.430	0.552	0.325	-0.335	-0.618	-0.178	0.429	0.740	0.330
	**pH_{CaCl2}**	0.045	0.084	-0.070	0.469	0.573	0.343	-0.292	-0.667	-0.248	0.3877	0.725	0.361
	**pH_{KCl}**	-0.011	0.041	-0.1380	0.409	0.532	0.310	-0.328	-0.6545	-0.215	0.413	0.720	0.314
	**EC**	0.226	0.343	0.387	0.446	0.634	0.585	-0.467	-0.881*	-0.599	0.516	0.720	0.529
	**CEC**	0.566	0.625	0.552	0.779	0.887*	0.798	-0.325	-0.993***	-0.799	0.427	0.798	0.806
	**HA**	0.336	0.311	0.476	-0.088	-0.185	0.062	0.244	0.242	-0.227	-0.302	-0.490	0.060
	**BCS**	0.323	0.383	0.251	0.666	0.789	0.625	-0.354	-0.897*	-0.564	0.459	0.830	0.632
	**SCS**	0.014	0.016	-0.216	0.452	0.533	0.290	-0.234	-0.628	-0.218	0.329	0.676	0.321
	**Cox**	0.7051	0.794*	0.841**	0.8809*	0.992***	0.951*	-0.378	-0.87	-0.761	0.503	0.844	0.950*
	**ρ_s**	-0.495	-0.658	-0.652	-0.607	-0.864	-0.867	0.707	0.741	0.481	-0.801	-0.955*	-0.769
	**Sand**	-0.424	-0.360	-0.115	-0.712	-0.684	-0.613	-0.028	0.878	0.871	-0.048	-0.433	-0.684
	**Silt**	0.440	0.373	0.097	0.721	0.679	0.605	0.061	-0.868	-0.877	0.016	0.410	0.685
	**Clay**	0.265	0.230	0.141	0.691	0.692	0.627	-0.040	-0.895*	-0.857	0.113	0.477	0.687

*** p<0.001, ** p<0.01, * p<0.05

Table S9. Correlation coefficients describing relationships between the Freundlich sorption coefficients, K_F ($cm^{3/n}\,\mu g^{1-1/n}\,g^{-1}$), half-lives, DT_{50} (days), and first-order dissipation rates, k_R ($days^{-1}$), obtained from the single compound test and values obtained from fitting measured concentrations of the parent compound and its metabolites during the degradation experiments with metazachlor, i.e., half-lives (DT_{50-1} and DT_{50-2}) and actual first-order dissipation rates ($k_{DR1-actual}$ and $k_{DR2-actual}$) for metazachlor and its metabolites, respectively.

		K_F			DT_{50}			k_R		
		**MET**	**OA**	**ESA**	**MET**	**OA**	**ESA**	**MET**	**OA**	**ESA**
DT_{50-1}	**MET**	-0.368			0.691					
DT_{50-2}	**OA (M04)**		-0.498			0.618				
DT_{50-2}	**ESA (M08)**			-0.750			0.646			
k_{DR1}	**MET**	0.404						0.858		
k_{DR2}	**OA (M04)**		0.764						0.993***	
k_{DR2}	**ESA (M08)**			0.785						0.650

		DT_{50-1}		DT_{50-2}		k_{DR1}		k_{DR2}	
		**MET**	**OA (M04)**	**ESA (M08)**	**M09**	**MET**	**OA (M04)**	**ESA (M08)**	**M09**
DT_{50-2}	**OA (M04)**	0.849							
	**ESA (M08)**	0.522	0.485						
	**M09**	0.758	0.834	0.845					
	**M11**	-0.319	-0.756	-0.200	-0.498				
k_{DR2}	**OA (M04)**					0.988**			
	**ESA (M08)**					0.771	0.846		
	**M09**					0.975**	0.996***	0.883*	
	**M11**					-0.353	-0.399	-0.152	-0.377

*** p<0.001, ** p<0.01, * p<0.05

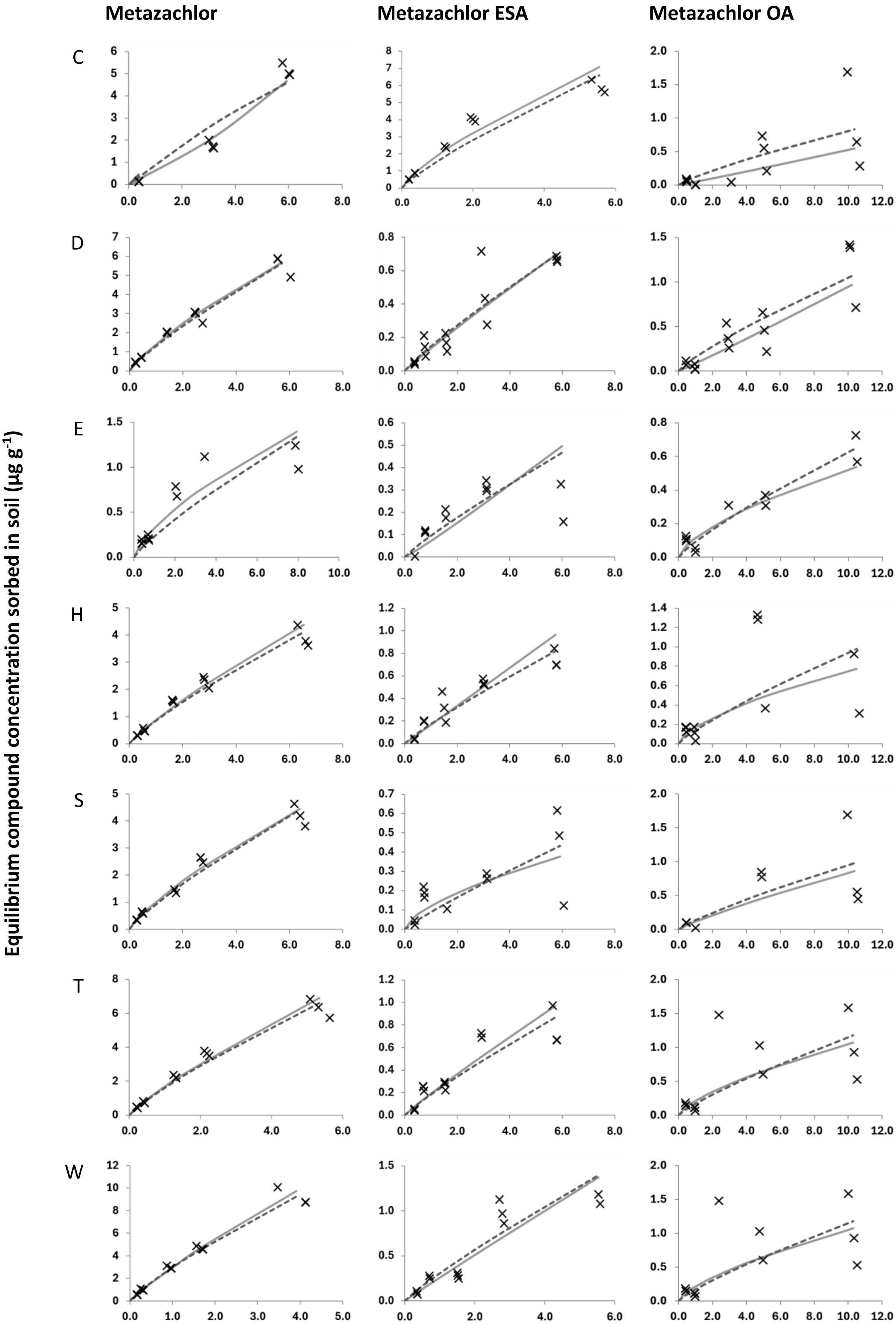

Metazachlor
Metazachlor ESA
Metazachlor OA
C
D
E
H
S
T
W
Equilibrium compound concentration sorbed in soil (µg g-1)

X

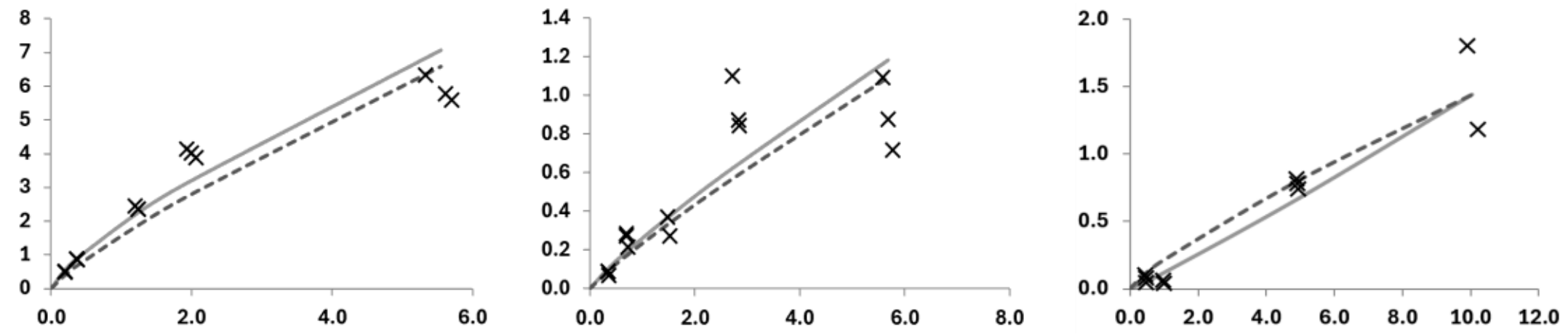

Equilibrium compound concentration in solution (μg cm^{-3})

Figure S1. Freundlich sorption isotherms for compounds (metazachlor, metazachlor OA, and metazachlor ESA) and soils (C – GP, D – HCh, E – AR, H – HCa-A, S – HL, T – HCa-B, W – DCa, and X – SChS,): experimental data (data-points), initially fitted with variable n value (dashed line), and fitted with the average n value for each compound (solid line).

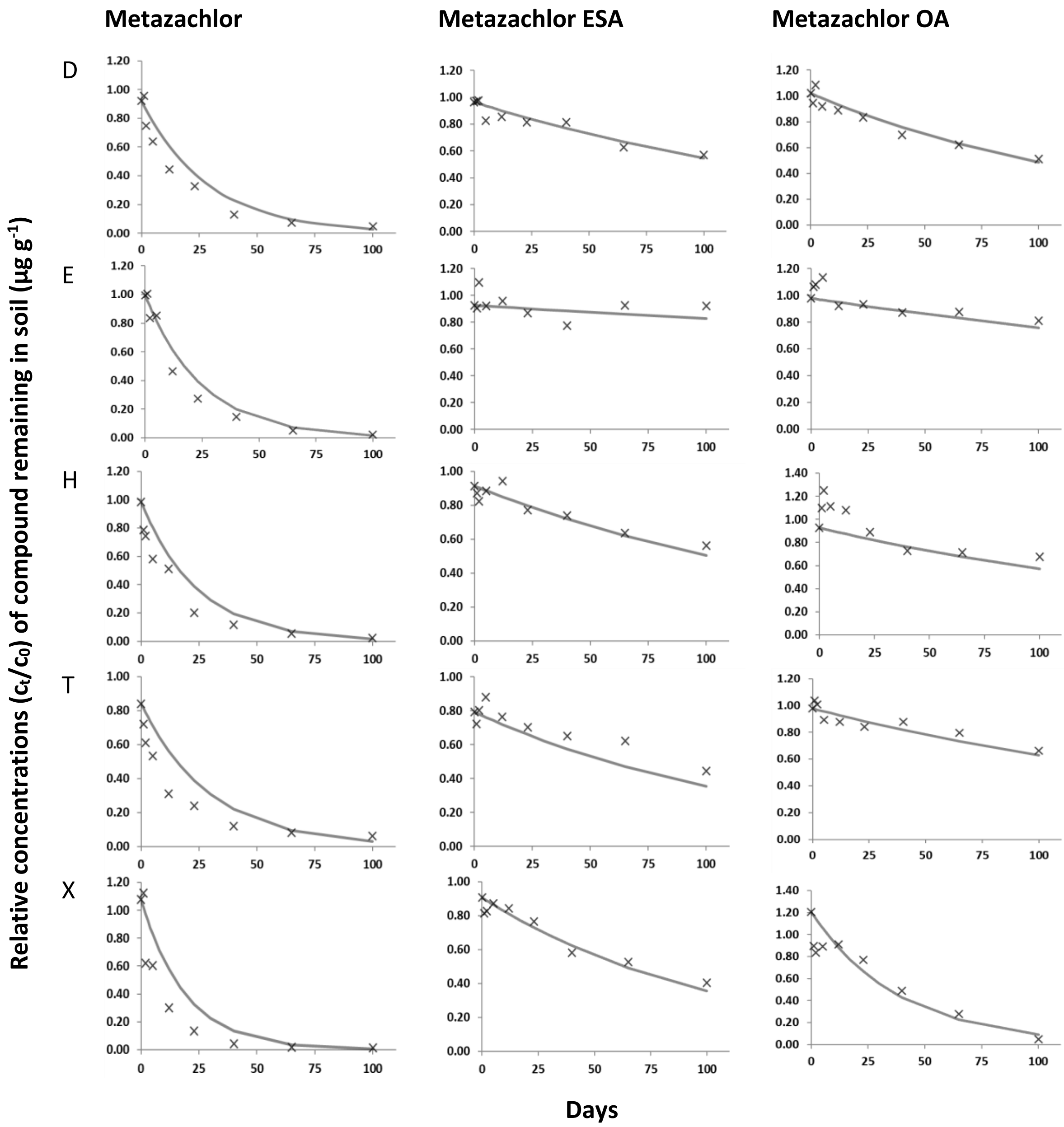

Figure S2. Kinetic plots to calculate the first order rate constants of compounds (metazachlor, metazachlor OA, and metazachlor ESA) and soils (D – HCh, E – AR, H – HCa-A, T – HCa-B, and X – SChS): experimental average values (data-points) and fitted dissipation trends (solid line)

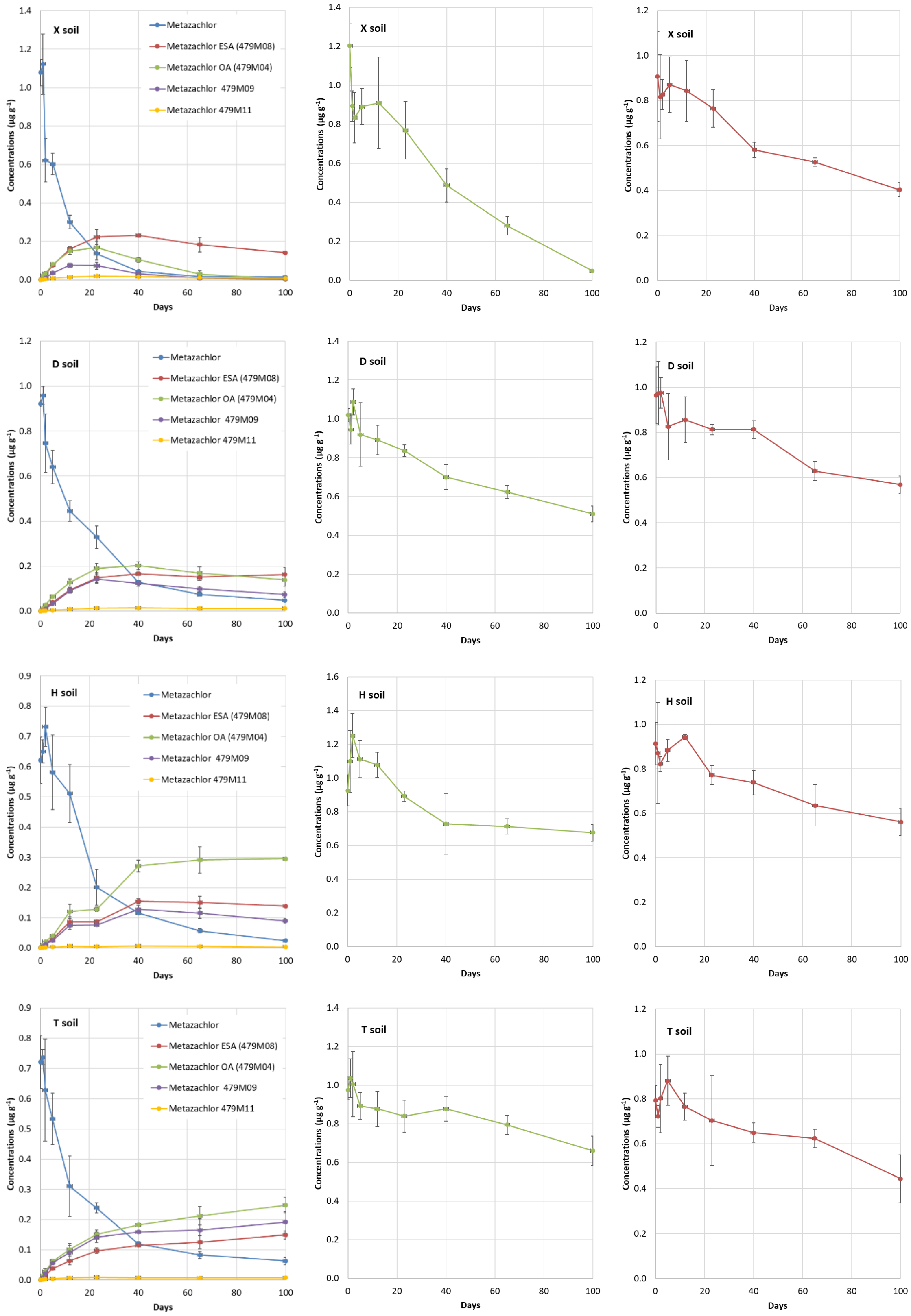

X soil
D soil
H soil
T soil
Metazachlor
Metazachlor ESA (479M08)
Metazachlor OA (479M04)
Metazachlor 479M09
Metazachlor 479M11
Concentrations (µg g⁻¹)
Days

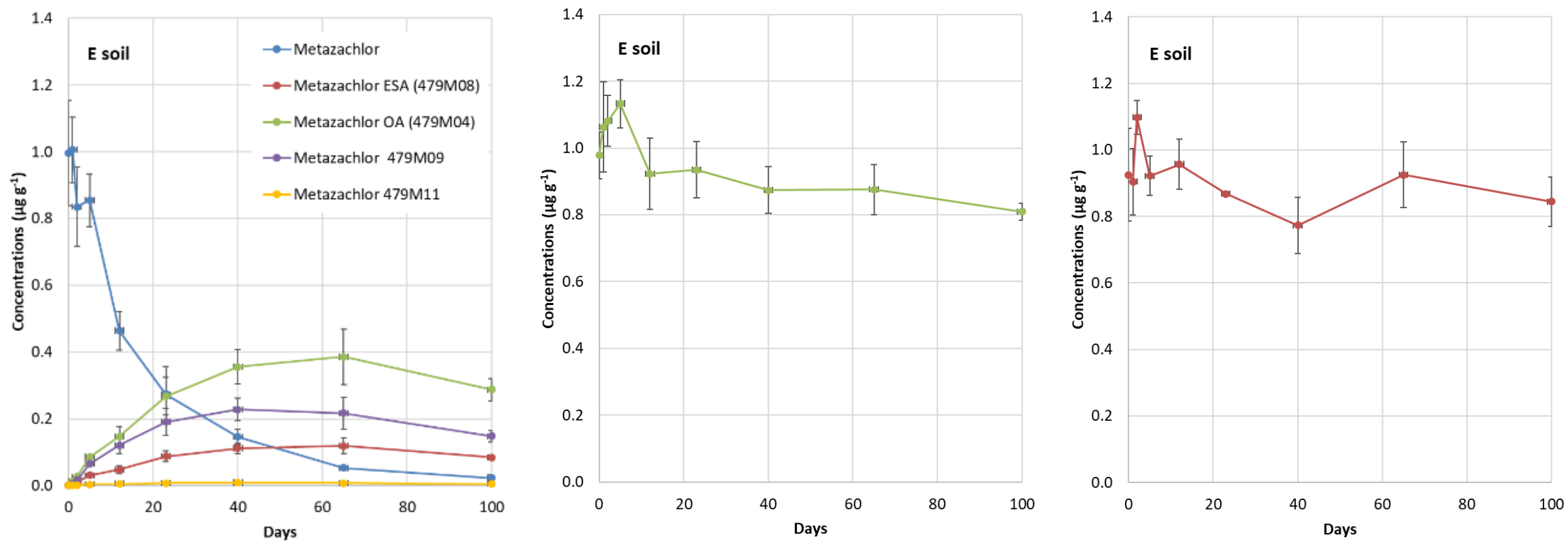

Fig. S3. Measured concetrations of metazachlor and ints metabolites during the degradation experiment with the parent compound metazachlo (left), metazachlor OA (midle) and metazachlor ESA (right) in different soils (D – HCh, E – AR, H – HCa-A, T – HCa-B, and X – SChS).
